# Association Between Low Muscle Mass and Gastric Hyperplastic and Inflammatory Polyps in Chinese Asymptomatic Adult Males

**DOI:** 10.3389/fmed.2022.861065

**Published:** 2022-05-06

**Authors:** Na Wang, Mengjun Chen, Chunjing Lin, Weihong Lin

**Affiliations:** ^1^Health Care Center, The First Affiliated Hospital of Wenzhou Medical University, Wenzhou, China; ^2^Department of Gastroenterology, The First Affiliated Hospital of Wenzhou Medical University, Wenzhou, China

**Keywords:** ASM/BMI, gastric polyps, Chinese asymptomatic adult males, BIA, upper gastrointestinal endoscopy, LMM

## Abstract

**Background:**

Gastric polyp is an abnormally proliferative or neoplastic growth of the gastric mucosa, with a degree of tendency to transform into gastric cancer. Lack of physical activity that is significantly related to low muscle mass (LMM) and muscle strength has been identified to be associated with gastric polyps. In this study, we examine the association of LMM and different histological types of gastric polyps among Chinese asymptomatic adult males.

**Methods:**

In total, 1,742 male adults undergoing bioelectrical impedance analysis and upper gastrointestinal endoscopies were included. Univariate and multivariate logistic regression models were used to analyze the effect of LMM on the risk of gastric polyps and different histological types.

**Results:**

By univariate and multivariate analyses, LMM (OR: 1.689, 95%CI: 1.046–2.726, *p* = 0.032) kept independent effect on risk of gastric polyps. When ratio of appendicular skeletal muscle mass to body mass index(ASM/BMI) was brought into the analyses, it was identified to be negatively correlated with occurrence of gastric polyps (OR: 0.744, 95%CI: 0.566–0.977, *p* = 0.033). For different pathological types, LMM showed different effect on occurrence of gastric polyps. LMM was an independent indicator for hyperplastic and inflammatory polyps (OR: 2.378, 95%CI: 1.288–4.389, *p* = 0.006), rather than fundic gland polyps (OR: 1.013, 95%CI: 0.473–2.173, *p* = 0.973).

**Conclusion:**

In general, LMM was an independent indicator for hyperplastic and inflammatory polyps occurrence in Chinese asymptomatic adult males.

## Introduction

Gastric polyp is an abnormally proliferative or neoplastic growth of the gastric mucosa, protruding into the lumen of stomach (1), with a degree of tendency to transform into gastric cancer (2). The incidence of gastric polyps varies between 1 and 6% in individuals who undergo upper gastrointestinal endoscopies (3–6). The most common subtypes of gastric polyps are fundic gland polyp, hyperplastic or inflammatory polyp, and adenomatous polyp (7).

A number of variables have been identified to have an impact on the risk of gastric polyps, such as age, gender, the incidence of Helicobacter pylori infection, use of proton pump inhibitors (PPIs), and physical activity (8–10). Females have a higher risk of gastric polyps (11, 12), so males with gastric polyps have received little attention. Additionally, research by Wang et al. (10) proposed that lack of regular exercise was significantly associated with gastric polyps. Reduction in physical activity was significantly related to low muscle mass (LMM), namely sarcopenia (13). It was speculated that LMM might be associated with gastric polyps. However, there were no such research about the connection between LMM and gastric polyps. To address this issue, the aim of this study was to determine whether LMM was related to gastric polyps and different histological types in Chinese adult males.

## Methods

We conducted a cross-sectional study of Chinese adult males having a health-check in the First Affiliated Hospital of Wenzhou Medical University between January 2018 and June 2018.

### Study Population

Inclusion criteria included: (1) males aged 18 years or older and (2) individuals who underwent bioelectrical impedance analysis (BIA) and upper gastrointestinal endoscopies from January to June 2018.

Exclusion criteria included: (1) patients with histories of PPIs administration for more than 1 year (17 individuals were eliminated from the study), (2) patients with histories of stomach surgeries (10 individuals), and (3) patients who were diagnosed with gastric cancers after upper gastrointestinal endoscopies (6 individuals).

### Data Collection

Baseline characteristic were collected by predesigned questionnaires, including age, gender, alcohol and smoking consumption, comorbidities, and histories of medication and surgery.

Smoking status was classified into 3 types: non-smoker (has never smoked before), past smoker (has quit for at least 2 yeas), and current smoker (is still smoking until the day of health-check or quit < 2 years). Males with alcohol consumption >140 g per week were regarded as heavy drinkers. Comorbidities were obtained by questionnaire and measurement, including hypertension (HTN), diabetes mellitus (DM), dyslipidemia, and metabolic syndrome (MS). The blood pressure measurement, blood detection, and ^13^C-urea breath test were performed on the morning of the health-check. The following blood measurements were tested: serum lipid profile, fasting blood glucose, and glycated hemoglobin.

The BIA and upper gastrointestinal endoscopy were performed in each individual. The appendicular skeletal muscle mass (ASM, kg) was measured by BIA (InBody770; InBody Korean Inc.). The ASM (kg)/BMI (kg/m^2^) was calculated to evaluate the muscle mass. According to the Foundation for the National Institutes of Health (FNIH) (14, 15), males with ASM/BMI <0.789 m^2^ were regarded as having low skeletal muscle mass. The endoscopic characteristics of polyps were recorded and analyzed, including existence, location, number, and size of pathological diagnosis. *Helicobacter Pylori* infection was detected by histopathological result of biopsied stomach specimen or ^13^C-urea breath test.

### Statistical Analysis

Category variables were compared by Pearson χ^2^ tests and displayed as frequencies (percentages). The normal distribution of continuous variables was tested by Shapiro–Wilk test. Continuous variables were compared by Wilcox test and displayed as median (range). Univariate and multivariate logistic regression analyses were performed to determine the effect of LMM on gastric polyp occurrence, containing different pathological types. Variables, including age, smoking status, heavy drinking status, diabetes mellitus, hypertension, dyslipidemia, metabolic syndrome, Helicobacter Pylori infection, and LMM, were analyzed by univariate analysis. Variables with *p* < 0.1 in univariate analysis were incorporated into the subsequent multivariate analysis. In addition, we compared ASM/BMI between individuals with and without gastric polyps by Wilcox test.

All statistical analyses were carried out using R version 3.6.1 (https://www.r-project.org/). All analyses were two-tailed, with *p* < 0.05 considered significant.

## Results

### Baseline Characteristics

We included 1,742 males, 1,487 of whom were in the group without LMM and 255 of them in the group with LMM (Table 1). The median of ASM/BMI was 0.903, range from 0.555 to 1.934. One hundred and ten males were diagnosed with gastric polyps (Table 2), with an incidence rate of 6.31%. There were three pathological types: fundic gland polyps (*N* = 54), hyperplastic and inflammatory polyps (*N* = 53), and adenomatous polyps (*N* = 1). The histology was not available on two individuals because of unsuitable biopsy specimens.

**Table 1:** Baseline characteristics between individuals with and without low skeletal muscle mass [median (range) or n (%)].

**Variables**	**Total (*N* = 1,742)**	**Individuals without LMM (*N* = 1,487)**	**Individuals with LMM (*N* = 255)**	***P*-value**
Age (years)	46 (18–84)	45 (18–78)	50 (21–84)	<0.001
**Smoking** (%)				<0.001
Never	1172 (67.28)	1020 (68.59)	152 (59.61)	
Past	20 (1.15)	12 (0.81)	8 (3.14)	
Current	550 (31.57)	455 (30.60)	95 (37.25)	
**Heavy drinker (%)**				0.429
Yes	316 (18.14)	265 (17.82)	51 (20.00)	
No	1426 (81.86)	1222 (82.18)	204 (80.00)	
**Diabetes mellitus (%)**				<0.001
Yes	179 (10.28)	134 (9.01)	45 (17.65)	
No	1563 (89.72)	1353 (90.99)	210 (82.35)	
**Hypertension (%)**				<0.001
Yes	594 (34.10)	470 (31.61)	124 (48.63)	
No	1148 (65.90)	1017 (68.39)	131 (51.37)	
**Dyslipidemia (%)**				0.014
Yes	1147 (65.84)	959 (64.49)	188 (73.73)	
No	591 (33.93)	524 (35.24)	67 (26.27)	
Missing	4 (0.23)	4 (0.27)	0 (0.00)	
**Metabolic syndrome (%)**				<0.001
Yes	312 (17.91)	230 (15.47)	82 (32.16)	
No	1430 (82.09)	1257 (84.53)	173 (67.84)	
**Helicobacter Pylori infection (%)**				0.868
Yes	784 (45.01)	666 (44.79)	118 (46.27)	
No	939 (53.90)	804 (54.07)	135 (52.94)	
Missing	19 (1.09)	17 (1.14)	2 (0.78)	

**Table 2:** Baseline characteristics according to different pathological type of gastric polyps [median (range) or n (%)].

**Variables**	**Total (*N* = 107)**	**Fundic gland polyps (*N* = 54)**	**Hyperplastic and inflammatory polyps (*N* = 53)**	***P*-value**
Age (years)	49.0 (25.0–84.0)	48.5 (25.0–84.0)	49.0 (29.0–78.0)	0.945
**Smoking (%)**				0.558
Never	73 (68.22)	38 (70.37)	35 (66.04)	
Past	2 (1.86)	0 (0.00)	2 (3.77)	
Current	32 (29.91)	16 (29.63)	16 (30.19)	
**Heavy drinker (%)**				0.797
Yes	17 (15.89)	8 (14.81)	9 (16.98)	
No	90 (84.11)	46 (85.19)	44 (83.02)	
**Diabetes mellitus (%)**				1.000
Yes	11 (10.28)	6 (11.11)	5 (9.43)	
No	96 (89.72)	48 (88.89)	48 (90.57)	
**Hypertension (%)**				0.173
Yes	45 (42.06)	19 (35.19)	26 (49.06)	
No	62 (57.94)	35 (64.81)	27 (50.94)	
**Dyslipidemia (%)**				0.605
Yes	72 (67.29)	38 (70.37)	34 (64.15)	
No	34 (31.78)	16 (29.63)	18 (33.96)	
Missing	1 (0.93)	0 (0.00)	1 (1.89)	
**Metabolic syndrome (%)**				0.639
Yes	22 (20.56)	10 (18.52)	12 (22.64)	
No	85 (79.44)	44 (81.48)	41 (77.36)	
**Helicobacter Pylori infection (%)**				0.012
Yes	25 (23.36)	7 (12.96)	18 (33.96)	
No	82 (76.64)	47 (87.04)	35 (66.04)	
**Low muscle mass (%)**				0.067
Yes	24 (22.43)	8 (14.81)	16 (30.19)	
No	83 (77.57)	46 (85.19)	37 (69.81)	
**Polyp location (%)**				<0.001
Antrum	10 (9.35)	0 (0.00)	10 (18.87)	
Body	60 (56.07)	37 (68.52)	23 (43.40)	
Fundus	18 (16.82)	13 (24.07)	5 (9.43)	
Polysite	9 (8.41)	3 (5.56)	6 (11.32)	
Other sites	10 (9.35)	1 (1.85)	9 (16.98)	
**Number of polyps**			
Single	77 (71.96)	38 (70.37)	39 (73.58)	0.830
Multiple^*^	30 (28.04)	16 (29.63)	14 (26.42)	
**Polyp size (cm)**				
<0.5	89 (83.18)	46 (85.19)	43 (81.13)	0.028
≥0.5	14 (13.08)	4 (7.41)	10 (18.87)	
undefined	4 (3.74)	4 (7.41)	0 (0.00)	

* *In individuals with multiple polyps, size was based on the largest polyp*.

### Univariate and Multivariate Analyses for Gastric Polyps

As Table 3 showed, variables including HTN (odds ratio [OR]: 1.523, 95% confidence interval [CI]: 1.021–2.272, *p* = 0.039), *Helicobacter Pylori* infection (OR: 0.339, 95%CI: 0.216–0.534, *p* < 0.001), and LMM (OR:1.689, 95%CI: 1.046–2.726, *p* = 0.032) had an independent effect on the risk of gastric polyps.

**Table 3:** Univariate and multivariate analyses for gastric polyps.

**Variables**	**Univariate analysis**		**Multivariate analysis**	
	**Crode OR (95% CI)**	***P*-value**	**Adjust OR (95% CI)**	***P*-value**
Age, ≥50 vs. <50	1.511 (1.025–2.227)	0.037	1.309 (0.875–1.959)	0.191
**Smoking (%)**				
Past vs. never	1.649 (0.375–7.240)	0.508		
Current vs. never	0.978 (0.643–1.487)	0.916		
Heavy drinker, yes vs. no	0.876 (0.520–1.474)	0.618		
Diabetes mellitus, yes vs. no	1.184 (0.649–2.158)	0.582		
Hypertension, yes vs. no	1.540 (1.042–2.276)	0.030	1.523 (1.021–2.272)	0.039
Dyslipidemia, yes vs. no	1.146 (0.755–1.741)	0.522		
Metabolic syndrome, yes vs. no	1.378 (0.867–2.192)	0.175		
Helicobacter Pylori infection, yes vs. no	0.349 (0.222–0.548)	<0.001	0.339 (0.216–0.534)	<0.001
Low muscle mass, yes vs. no	1.792 (1.124–2.861)	0.014	1.689 (1.046–2.726)	0.032

When ASM/BMI was brought into the analyses as a continuous variable (Supplementary Table 1), it was identified to be negatively correlated with the occurrence of gastric polyps in univariate (OR: 0.727, 95%CI: 0.557–0.948, *p* = 0.019) and multivariate analyses (OR: 0.744, 95%CI: 0.566–0.977, *p* = 0.033).

### Univariate and Multivariate Analyses for Different Histological Types of Gastric Polyps

In univariate analysis (Supplementary Table 2), only *Helicobacter Pylori* infection (OR: 0.171, 95%CI: 0.077–0.380, *p* < 0.001; Supplementary Table 2) showed an association with risk of fundic gland polyps. It was inferred that LMM (OR: 1.013, 95%CI: 0.473–2.173, *p* = 0.973; Table 4) had no significant effect on the occurrence of fundic gland polyps.

**Table 4:** Logistic Regression Analyses for the different histologic types in gastric polyps.

**LMM**	**Effect of LMM for fundic gland polyps**		**Effect of LMM for hyperplastic and inflammatory polyps**	
	**OR (95% CI)**	***p*-value**	**OR (95% CI)**	***p*-value**
Univariate analysis	1.013 (0.473–2.173)	0.973	2.620 (1.435–4.784)	0.002
Multivariate analysis	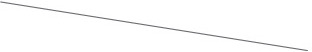	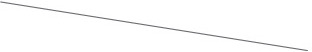	2.378 (1.288–4.389)	0.006

In univariate and multivariate analyses (Supplementary Table 3), compared with the group without LMM, there was an 137.8% rise in the risk of hyperplastic and inflammatory polyps in individuals with LMM (OR: 2.378, 95%CI: 1.288–4.389, *p* = 0.006; Table 4).

### Different ASM/BMI in Individuals With and Without Gastric Polyps

**Figure 1:**
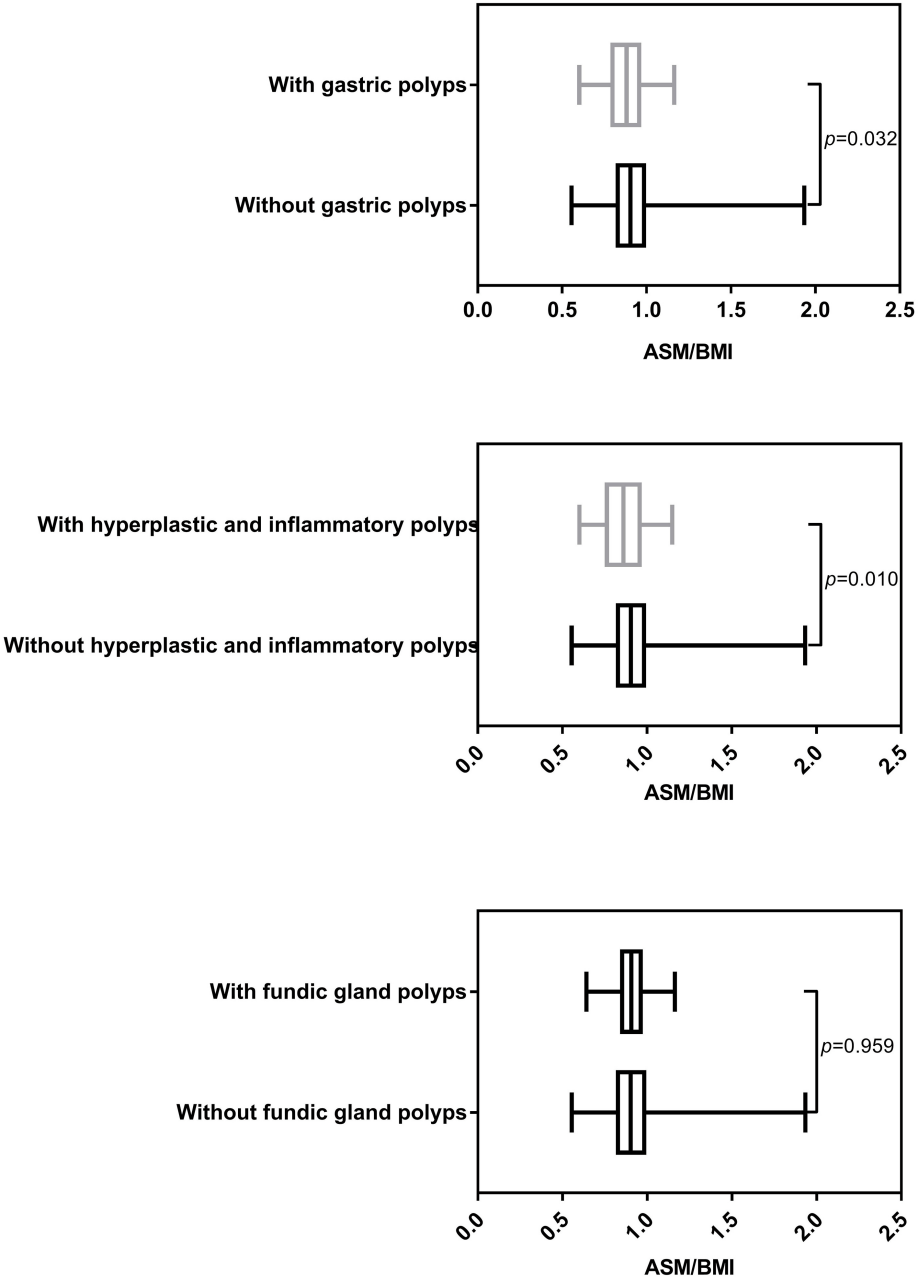
Different ASM/BMI of individuals with and without gastric polyps (including different histological types).

As Figure 1 showed, individuals with and without gastric polyps displayed significantly different ASM/BMI (*p* = 0.032). Individuals with hyperplastic and inflammatory polyps showed relatively lower ASM/BMI (*p* = 0.010) compared to individuals without hyperplastic and inflammatory polyps. However, this association was not observed in fundic gland polyps (*p* = 0.959).

**Figure 2:**
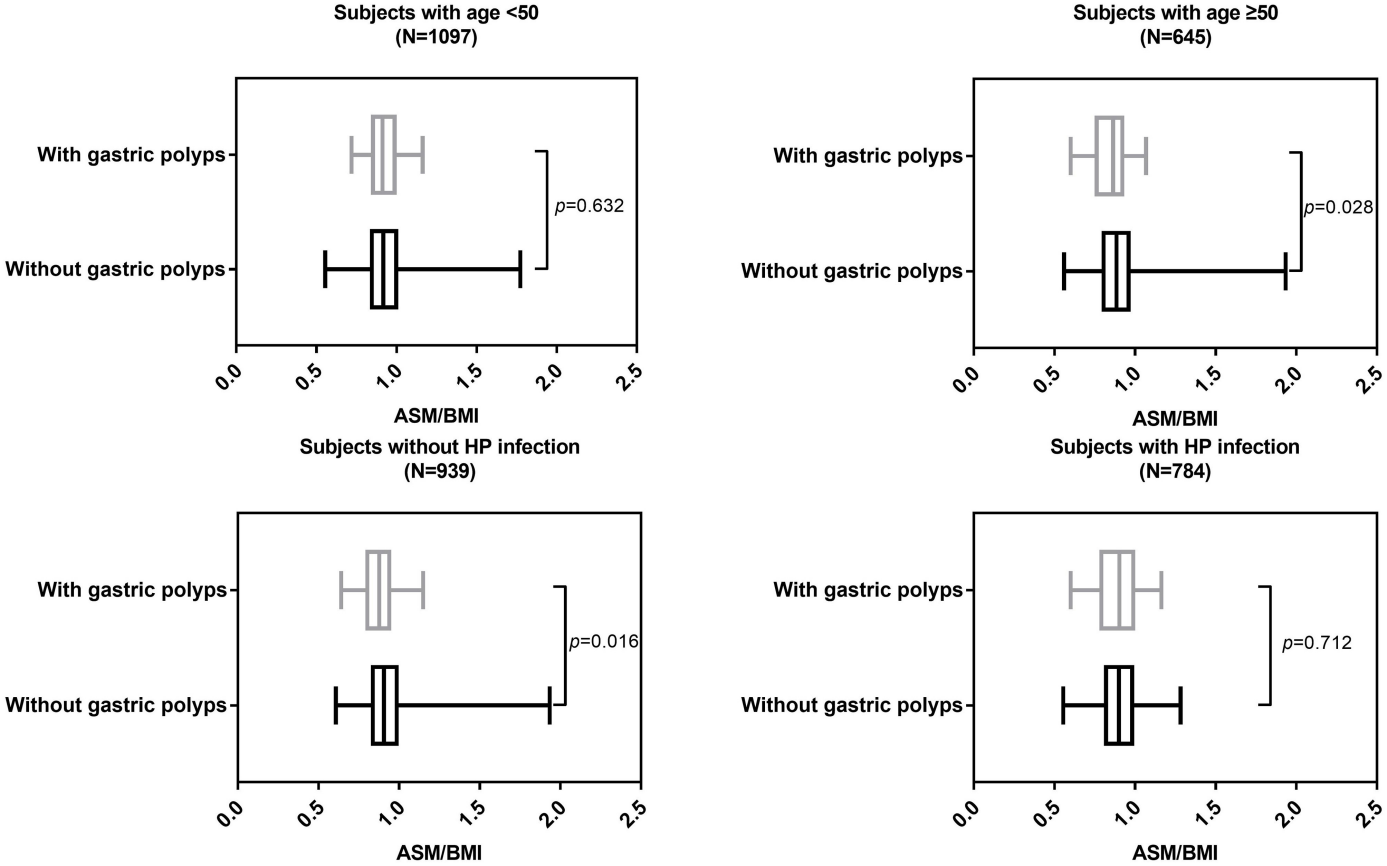
ASM/BMI of individuals with and without gastric polyps in different subgroups.

**Figure 3:**
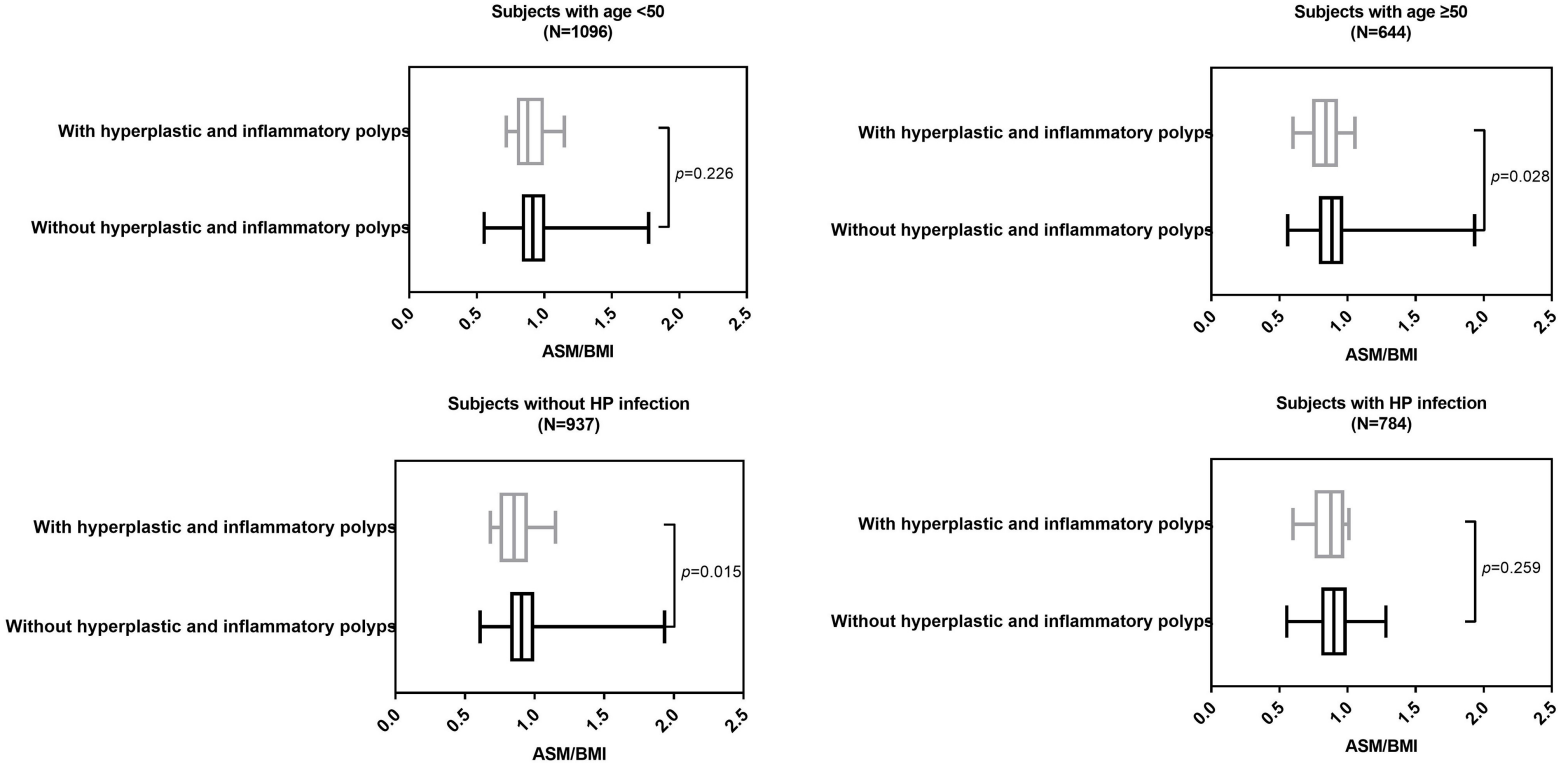
ASM/BMI of individuals with and without hyperplastic and inflammatory polyps in different subgroups.

In individuals with age ≥ 50 (*p* = 0.028) and individuals without HP infection (*p* = 0.016), ASM/BMI was still significantly different between individuals with and without gastric polyps (Figure 2). Similarly, in individuals with age ≥ 50 (*p* = 0.028) and individuals without HP infection (*p* = 0.015), ASM/BMI was significantly higher in individuals without hyperplastic and inflammatory polyps (Figure 3).

## Discussion

In this study, we found an association between LMM and increased occurrence of hyperplastic and inflammatory polyps in asymptomatic males. Up to now, there has been no research concerning the relation between reduced muscle mass and occurrence of gastric polyps. However, the inverse associations between physical activity and the presence of colorectal polyps, adenomas, and even cancer have been previously reported (16–21). A study by Wang et al. (10) mentioned that lack of physical exercise was significantly associated with gastric polyps. Physical inactivity was a major cause of sarcopenia, which manifested as reduced muscle mass and strength (22). There might be an underlying connection between low muscle mass, gastric polyps, and physical inactivity. A relevant mechanism study about the relationship is needed in the future.

The population enrolled in this study was Chinese asymptomatic adult males. Moreover, we included 1,100 females and discovered that there was no connection between LMM and gastric polyps with *p*-value of 0.168 (Supplementary Table 4). Sarcopenia and gastric polyps are more common in females (23, 24). But the correlation between low muscle mass and gastric polyps was found to exist in males in this study. It was inferred that sex hormones were responsible for the gender-specific difference. A further study about the underlying mechanism of gender-specific correlation between low muscle mass and gastric polyps is needed in the future.

The pathological types of gastric polyps in our study found to be associated with low muscle mass was hyperplastic or inflammatory polyps. It was caused by hyperplasia of foveolar cells accompanied by their increased exfoliation (25, 26). Gastric hyperplastic or inflammatory polyps commonly reside in gastric mucosa with longstanding Helicobacter Pylori infection-associated atrophy or metaplasia (25). In a study by Baeg et al. (27) it was discovered that Helicobacter Pylori eradication treatment increased ghrelin secretion and reduced the risk of low muscle mass. Thus, the correlation between gastric hyperplastic or inflammatory polyps and LMM might be Helicobacter Pylori infection-associated.

Muscle mass, which could be easily quantitatively detected by BIA or dual X-ray absorptiometry (DXA), is cost-effective and of great importance for the diagnosis of sarcopenia (14). It was necessary to detect the muscle mass before gastrointestinal endoscopies, which is predictive for hyperplastic and inflammatory polyps. In addition, enhanced physical exercise might be a preventive action to avoid possible gastric polyps to some extent. However, further prospective studies are required to determine the effect of physical activity on risk of gastric polyps.

## Study Strengths and Limitations

It was cross-sectional study with large sample size, and there was a new-found association between low muscle mass and hyperplastic or inflammatory polyps. Detection of muscle mass prior to gastrointestinal endoscopies could help doctors with psychological preparation for corresponding removal of polyps.

Yet, there were some limitations to our study: (1) cross-sectional nature of the research, which could not prove causality; (2) a single-center analysis with ineluctable selective bias; (3) a lack of data of muscle strengthening, which caused failure to diagnosis individuals with sarcopenia; and (4) a lack of data on physical activity/exercise, which mediated the process of low muscle mass and gastric polyps to some extent.

## Conclusion

In general, low muscle mass was a potential indicator for predicting occurrence of hyperplastic or inflammatory polyps, rather than fundic gland polyps. The ASM/BMI was negatively correlated with the occurrence of hyperplastic or inflammatory polyps.

## Data Availability Statement

The original contributions presented in the study are included in the article/Supplementary Material, further inquiries can be directed to the corresponding author/s.

## Ethics Statement

The study was approved by the Institutional Review Board (IRB) of The First Affiliated Hospital of Wenzhou Medical University. Due to its cross-sectional nature, consent was waived by IRB and the data was anonymous. All data was extracted from electronic medical records and analyzed in accordance with the principles of the Declaration of Helsinki.

## Author Contributions

CL: data curation. MC: methodology. NW: writing—original draft. WL: writing—review and editing. All authors contributed to the article and approved the submitted version.

## Conflict of Interest

The authors declare that the research was conducted in the absence of any commercial or financial relationships that could be construed as a potential conflict of interest.

## Publisher's Note

All claims expressed in this article are solely those of the authors and do not necessarily represent those of their affiliated organizations, or those of the publisher, the editors and the reviewers. Any product that may be evaluated in this article, or claim that may be made by its manufacturer, is not guaranteed or endorsed by the publisher.
